# Emergence of three general practitioner contracting-in models in South Africa: a qualitative multi-case study

**DOI:** 10.1186/s12939-018-0830-0

**Published:** 2018-10-05

**Authors:** Linda Mureithi, James Michael Burnett, Adam Bertscher, René English

**Affiliations:** 1 Health Systems Research Unit, Health Systems Trust, 1st Floor Block B, Aintree Park, Kenilworth, Cape Town, 7700 South Africa; 2 School of Public Health and Family Medicine, University of Cape Town, Cape Town, South Africa

**Keywords:** Universal health care, Public private sector, Low-and-middle-income countries, South Africa, Contracting, Contracting-in, Primary health care, General practitioner, Non-state provider, Health policy and systems research

## Abstract

**Background:**

The general practitioner contracting initiative (GPCI) is a health systems strengthening initiative piloted in the first phase of national health insurance (NHI) implementation in South Africa as it progresses towards universal health coverage (UHC). GPCI aimed to address the shortage of doctors in the public sector by contracting-in private sector general practitioners (GPs) to render services in public primary health care clinics. This paper explores the early inception and emergence of the GPCI. It describes three models of contracting-in that emerged and interrogates key factors influencing their evolution.

**Methods:**

This qualitative multi-case study draws on three cases. Data collection comprised document review, key informant interviews and focus group discussions with national, provincial and district managers as well as GPs (*n* = 68). Walt and Gilson’s health policy analysis triangle and Liu’s conceptual framework on contracting-out were used to explore the policy content, process, actors and contractual arrangements involved.

**Results:**

Three models of contracting-in emerged, based on the type of purchaser: a centralized-purchaser model, a decentralized-purchaser model and a contracted-purchaser model. These models are funded from a single central source but have varying levels of involvement of national, provincial and district managers. Funds are channelled from purchaser to provider in slightly different ways. Contract formality differed slightly by model and was found to be influenced by context and type of purchaser. Conceptualization of the GPCI was primarily a nationally-driven process in a context of high-level political will to address inequity through NHI implementation. Emergence of the models was influenced by three main factors, flexibility in the piloting process, managerial capacity and financial management capacity.

**Conclusion:**

The GPCI models were iterations of the centralized-purchaser model. Emergence of the other models was strongly influenced by purchaser capacity to manage contracts, payments and recruitment processes. Findings from the decentralized-purchaser model show importance of local context, provincial capacity and experience on influencing evolution of the models. Whilst contract characteristics need to be well defined, allowing for adaptability to the local context and capacity is critical. Purchaser capacity, existing systems and institutional knowledge and experience in contracting and financial management should be considered before adopting a decentralized implementation approach.

**Electronic supplementary material:**

The online version of this article (10.1186/s12939-018-0830-0) contains supplementary material, which is available to authorized users.

## Introduction

Universal health coverage (UHC) is a fundamental health system goal and a key target of the health-related sustainable development goal (SDG) [1–3]. In recent years South Africa (SA) arrived at a policy decision to progressively realize the attainment of health for all in SA in part through a 14-year phased introduction of UHC using National Health Insurance (NHI) as the financing mechanism [4].

Contracting private sector providers to render services to uninsured public sector patients was one of four streams of a “PHC re-engineering” strategy that sought to strengthen delivery of primary health care (PHC) services at district level in preparation for the future introduction of NHI [5]. An initiative, referred to in this paper as the ‘GP contracting initiative’ (GPCI), was designed to contract-in private sector General Practitioners (GPs) to render services in the form of time-bound sessions in public sector PHC facilities.

Health policy and systems research (HPSR) aims to explore the “what”, “how” and “why” of policy development and implementation [6]. Documenting the GPCI using existing health policy analysis frameworks, this paper provides detailed accounts of three models of contracting-in and lays a foundation for forthcoming publications that will provide further in-depth analysis on the GPCI. Comparing the different models that were piloted in different locales provides rich information that could potentially guide policymakers’ future strategies for engaging non-state actors in SA, as well as in other similar settings, as they progress towards UHC.

## Background

Defined as access to quality health services for all citizens [3, 7], UHC encompasses both provision of the full range of quality essential health services according to need and protection from financial hardship due to out-of-pocket payments for health services [3, 8].

SA has a two-tiered health care system that is comprised of a public sector, primarily funded through tax contributions, and a private sector funded through medical schemes (private health insurance), hospital care plans and out-of-pocket payments (OOPs) [9, 10]. This system results in inequitable access to care for the population. The impact of disparate health care financing and resourcing between the sectors is evident across economic, racial and geographical strata, [11, 12] and is illustrative of the inverse care law [13–17]. In 2014, total health expenditure accounted for 8.5% of Gross Domestic Product (GDP), with about half (4.3%) spent in the private sector that serves only 18.1% of the population [12, 18, 19]. In 2008, per capita spending by private medical schemes was found to be more than five times higher than in the public sector [9]. The costs of health care services, as well as spending, vary significantly between the two sectors.

Although voluntary private medical schemes mainly cater to high- and middle-income formal sector employees, the law requires that members receive a prescribed minimum benefit (PMB) package of health services [9, 10, 20]. Private health care providers are typically remunerated by medical schemes on a fee-for-service (FFS) basis, with scheme members incurring OOP expenditures for services not covered under the PMB package. The uninsured population is primarily dependent on the public sector for health services. However, evidence suggests that uninsured low-income workers frequently access private providers directly for primary care services such as general practitioner (GP) consultations [9, 10, 21] thus also being subject to OOP payments.

The introduction of NHI is an endeavor that is likely to involve significant health financing reforms aimed at pooling revenue to improve cross-subsidization; it also seeks to use economies of scale and strategic purchasing to achieve cost-efficiency. An NHI fund will eventually be established as a single-payer and single-purchaser to purchase health care services from a mix of private and public providers [22].

The first phase of SA’s effort to attain NHI focused on health systems strengthening aimed at improving the quality of health services in the public sector and addressing structural imbalances, including public sector human resource shortages [4, 23]. To this end, the Ministry of Health (MOH) developed a plan to achieve better population-based health care outcomes by strengthening the PHC services delivered through the District Health System [24]. Contracting private sector providers to render services to uninsured patients was one of four streams of “PHC re-engineering” [5]; General Practitioners (GPs) were the first cadre of health care professionals to be contracted through the GPCI. This study explores the early implementation of the initiative.

### The history of contracting health care providers in South Africa

Contracting of GPs into SA’s public sector has in fact been implemented for many years in various forms. Prior to 1994, the “Part Time District Surgeons” (PTDS) programme contracted private GPs to provide FFS PHC services in their own practices with the aim of providing access to care particularly in rural hard-to-reach geographical locations [25]. These contracts faced challenges, including administrative difficulties, and MOH concerns about quality of services and abuses of the system by doctors due to insufficient levels of oversight [25, 26]. These issues, coupled with patient concerns regarding quality of care and equity [25, 26], eventually resulted in the discontinuation of the programme after 1994.

The GPCI pilot commenced in 11 NHI pilot districts in early 2013, with the purpose of testing different models of contracting-in in various contexts. The policy intent of the GPCI is to address health care personnel shortages in the public sector, and specifically to increase access to quality health care in rural and isolated geographical areas. The national shortage of doctors has been well documented. With an estimated 76.7 doctors per 100,000 population in the country [27], SA seems to compare relatively favorably to the average physician density for other low-and-middle-income countries (LMICs) (80 per 100,000) [27]. However, notable disparities exist between the public and private sectors [28]. One analysis estimated that there were 25.1 GPs per 100,000 population in the public sector, compared to 92.5 per 100,000 population in the private sector in 2013 [29]. Therefore a key intention of the GPCI was to draw on the better-resourced private sector, to fill gaps in the public sector’s human resources. The GPCI can be described as a formal spoken policy. It was implemented as a pilot in order to identify implementation model(s) that could potentially be scaled up during the phasing in of NHI, and to identify best practices for future application. To date, however, the models have not been described in detail nor has any formal evaluation been conducted.

During the inception and early implementation of the GPCI, three distinct models of contracting-in emerged. Using existing health policy analysis frameworks, this paper seeks to (1) describe the three models of contracting-in, (2) describe the inception of GPCI and its introduction into the South African public health sector, and (3) identify key factors that influenced the early emergence of these three models, with a focus on contextual, contractual and actor-related factors.

## Methods

### Theoretical frameworks

We used Walt and Gilson’s health policy analysis triangle [30] as the primary analytical framework to explore the policy content, contexts, processes and actors involved in the development and early implementation of the GPCI, in order to describe how the three models emerged and which factors influenced their evolution and characteristics. To enable a more explicit examination of the contractual arrangements and actors involved in each of these models, we incorporated Liu’s conceptual framework on contracting-out [31] within the overarching Walt and Gilson framework. Although not specifically adapted for contracting-in, Liu’s framework guided our review of the features of a formal contract: type of services, contract formality and duration, provider selection, provider payment mechanisms, specification of performance requirements and characteristics of the purchaser and provider as actors, as well as how these changed over time [31].

### Research design

This paper draws on qualitative data that were collected as part of a larger mixed methods study exploring the implementation of the GPCI, the actors involved, and the interactions among them. The broader study involved analysis of secondary data and qualitative data collection. Secondary quantitative data were used to inform case selection for this qualitative multi case-study. A case study design enables the in-depth exploration of a phenomenon, such as the GPCI, within its context, and through a variety of perspectives using multiple sources of evidence [32, 33]. We selected multiple case studies to enable exploration of differences within and between cases (models) [33]. We purposively selected three case study districts to represent three of the nine GPCI pilot districts. The districts represented various contexts in which the GPCI piloting was taking place: one urban densely-populated well-resourced district, one rural sparsely-populated district, and one rural but relatively well-resourced district that represented a mid-point between the other two (Table 1). Primary qualitative data collected at national, provincial and district levels were used to describe the inception of the GPCI, the three types of contracting models that emerged and the factors that influenced the emergence of these models.

**Table 1:** Key characteristics of case study districts

	DISTRICT A	DISTRICT B	DISTRICT C
Contracting model			
Contracting model(s)	Decentralized-purchaser	Contracted-purchaser Centralized-purchaser	Contracted-purchaser Centralized-purchaser
Demographics			
Total population	595,542	3,165,745	718,549
Uninsured population	493,389	2,115,620	674,771
Percentage uninsured (%)	82.85	66.83	93.91
Population density	26 people/km^2^	503 people/km^2^	22 people/km^2^
Socio-economic			
Socio-economic quintile (SEQ)^a^	4	5	3
Rural vs. urban	Rural	Urban	Rural
Health status			
Crude death rate (per 1000 population)	3.6	4.7	4.6
HIV antenatal prevalence (15–49 years)	15.6	23.4	30.1
Incidence of TB (per 100,000 population)	806	351	511
Health service			
PHC utilization rate^b^	2.41	1.64	2.78
PHC nurse clinical workload^c^	25.3	36.7	34.6
PHC doctor clinical workload	26.5	29.1	34.5
No. of GPs contracted through model as of June 2016	14	87	29
Number of PHC health facilities	50	70	73

^a^The SEQ is derived from the South African Index of Multiple Deprivation (SAIMD). SAIMD is a composite indicator of socio-economic status developed from census data. It encompasses material, employment, educational and living environment deprivation. There are five SEQs based on a numeric SAIMD value with SEQ 1 representing the most deprived and 5 the least deprived

^b^PHC utilization rate is the rate at which PHC services are utilized by the catchment population. It represents the average number of visits per person per year in the catchment population with the denominator being a census-derived estimate. It is useful in determining the overall PHC utilization patterns and could be specifically relevant in tracking equity in health service utilization

^c^The PHC clinical workload is the average number of patients seen per health care worker (professional nurse or doctor) per clinical work day. These represent health care workers employed within the public sector as opposed to those contracted-in to provide services

In interviews with national-level policy-makers, the three models of contracting-in being piloted were revealed: centralized-purchaser, decentralized-purchaser and contracted-purchaser models. The contracting model thus became an additional key factor influencing the purposive selection of districts for study. Data were subsequently collected from Districts A, B and C. During data collection in the latter two districts it became apparent that two contracting models were being implemented concurrently in both. Hence the three districts no longer represented distinct cases as originally envisioned when the study was designed. The boundaries of the case studies were therefore redefined during the analysis phase to address the three contracting models. The three cases presented in this paper thus examine the three contracting models currently in existence, as presented in Table 1.

### Data sources

For the broader study, qualitative data comprised document review, key informant interviews (KIIs) and focus group discussions (FGDs) with national, provincial and district managers and general practitioners taking part in the GPCI. Documents reviewed included published and unpublished documents obtained from official websites and provided by key informants. These included policy documents relating to NHI, GPCI progress reports, contracts and job descriptions.

Participants for KIIs were purposively sampled at national, provincial and district levels based on their position, knowledge of and involvement in NHI generally and specifically GPCI policy formulation and implementation. Snowballing was used until saturation was reached. Purposive sampling was also used to obtain a range of perspectives across the various levels of the health system and contexts in order to obtain an in-depth understanding of this initiative. The research team engaged with the GPCI coordinator in each district to identify key stakeholders involved in district level implementation given that it was envisaged that the latter would differ by context.

Three FGDs were conducted with purposively selected district-level participants (including the District Manager and key District Health Management Team (DHMT) members) based on their active involvement in GPCI implementation. It was envisaged that different DHMT members would be responsible for different aspects of implementation of the GPCI, such as recruitment, contracting, placement, training and orientation, supervision, performance management and payment. The FGDs thus aimed to collect information on: the coordination of the GPCI at district level from the perspective of the DHMT, how the DHMT members interacted with each other to implement the initiative, challenges experienced and any gaps in DHMT capacity to oversee the contracting process. GPs for KIIs were selected through stratified random sampling. A list of all contracted GPs was obtained from each district. The GPs were stratified by sub-district and then randomly selected. A total of 56 KIIs and three FGDs were conducted. Seven respondents refused to participate, the reasons for which are unknown (Table 2).

**Table 2:** Profile of respondents and non-respondents by category

Category	Number of respondents (n)	Number of non-respondents (n)
KIIs		
National level managers	9	3
Provincial and district level managers	17	2
General practitioners	30	0
*Total number of respondents (KIIs)*	56	5
FGDs		
District FGD respondents	12	2
*Total number of respondents (FGDs)*	12	2
Total	68	7

To describe and explore the emergence of the models for this paper, we drew primarily on qualitative data from national, provincial and district-level KIIs and FGDs. Data from the GP interviews were used to corroborate data from managers’ describing how the models operate in practice.

### Data collection

KII and FGD guides were created to conduct the interviews and discussions. These guides were developed using key concepts identified in the literature and relevant theoretical frameworks described above [30, 31]. Questions focused on: participants’ roles in relation to the GPCI; policy origins and conceptualization; policy content; the implementation process, including influencing factors; and the actors involved, their experiences and understanding of the GPCI, and the relationships and interactions among them. The interview questions were developed relative to the role of each type of respondent. The interview guides were pilot tested prior to data collection.

All KIIs and FGDs were conducted in English as all respondents were conversant in English. KIIs were either conducted face-to-face or telephonically where face-to-face interviews were not possible. FGDs were all conducted face-to-face. Interviews were recorded on a digital analog recorder. Data were collected between June 2016 and May 2017 jointly by the four authors (LM, MB, AB and RE). Informed consent to conduct and record each interview was obtained from each participant. All interviews were transcribed verbatim. Transcripts were anonymized and imported into QSR NVIVO 11 for further coding.

### Data analysis

A preliminary deductive codebook was developed based on key factors in the two theoretical frameworks [30, 31]. Initial codes were based on key factors identified in Liu’s conceptual framework [31]. These were then grouped into four overarching groups (main codes) as per Walt and Gilson’s health policy analysis triangle: policy content, process, context and actors [30] as depicted in Table 3.

**Table 3:** Overview of codes used in analysis

	Policy content	Policy process	Actors	Context
Sub-codes	• Types of services • Contract formality • Contract duration • Provider selection • Provider payment mechanisms • Performance requirements • Motivation and incentive structures	• Extent of implementation • Process of engagement • Monitoring • Provider-purchaser behavior	• Purchaser type • Provider type • Provider/ purchaser capacity • Position (actor’s stance towards GPCI) • Provider/ purchaser individual factors • Provider/ purchaser level of power • Relationship between the provider and purchaser	• Structural contextual factors • Situational contextual factors • Cultural contextual factors • Exogenous/ international contextual factors

Data were coded and then summarized into matrices by theme to allow for comparison of themes across respondents and cases [34]. Findings were triangulated across respondents and with data obtained from document review. Data extracted from document review were also used to triangulate and develop a timeline of policy development, policy content, contract features, processes and actors involved. Divergent themes were discussed by the research team in-depth to explore nuances within and between the cases. Themes from the health policy analysis triangle [30] and Liu’s conceptual framework on contracting-out [31] were used to explore the factors influencing emergence of the contracting models. In addition, themes from the latter framework [31] were used to compare characteristics of contractual arrangements in the three models.

### Ethics

Ethical clearance for the study was obtained from the University of Cape Town Human Research Ethics Committee (HREC 189/2015) and the WHO Ethics Review Committee (ERC.0002661). In addition, permission to conduct data collection was obtained from the relevant Provincial Health Research Committees as per local research requirements. Participation in the study was voluntary and participants had the option to withdraw at any time. Informed consent was obtained from all participants prior to conducting the interviews. All identifiers were removed from transcripts to ensure participant confidentiality.

## Results

**Fig. 1:**
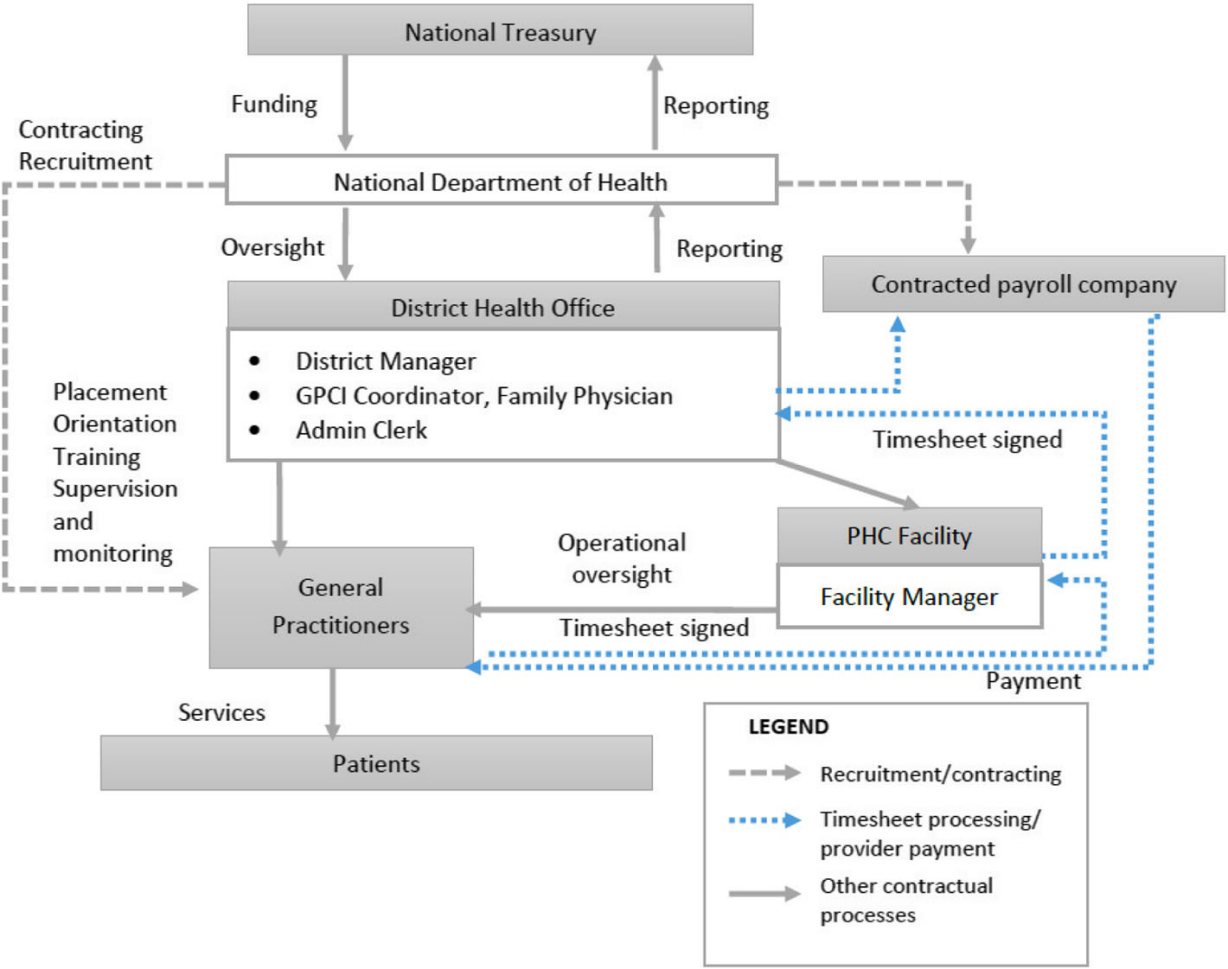
Centralized-purchaser model.The NDOH as the purchaser directly recruits and contracts GPs. Contracts are signed by a district manager (DM) an authorized signatory at NDOH. Placement, orientation, training, supervision and monitoring of GPs are done by staff at the district health office (DHO). GPs provide PHC services to patients attending PHC clinics with day-to-day oversight from a Facility Manager (FM). GPs are paid monthly by an external payroll company on behalf of the NDOH. This is effected on submission of a completed timesheet, signed and verified by the FM, GPCI Coordinator and DM. The DM is the final signatory required to effect payment. The DHO compiles and submits monthly and quarterly reports to the NDOH, containing information on the number of GPs appointed, hours worked and the estimated number of patients seen per hour

**Fig. 2:**
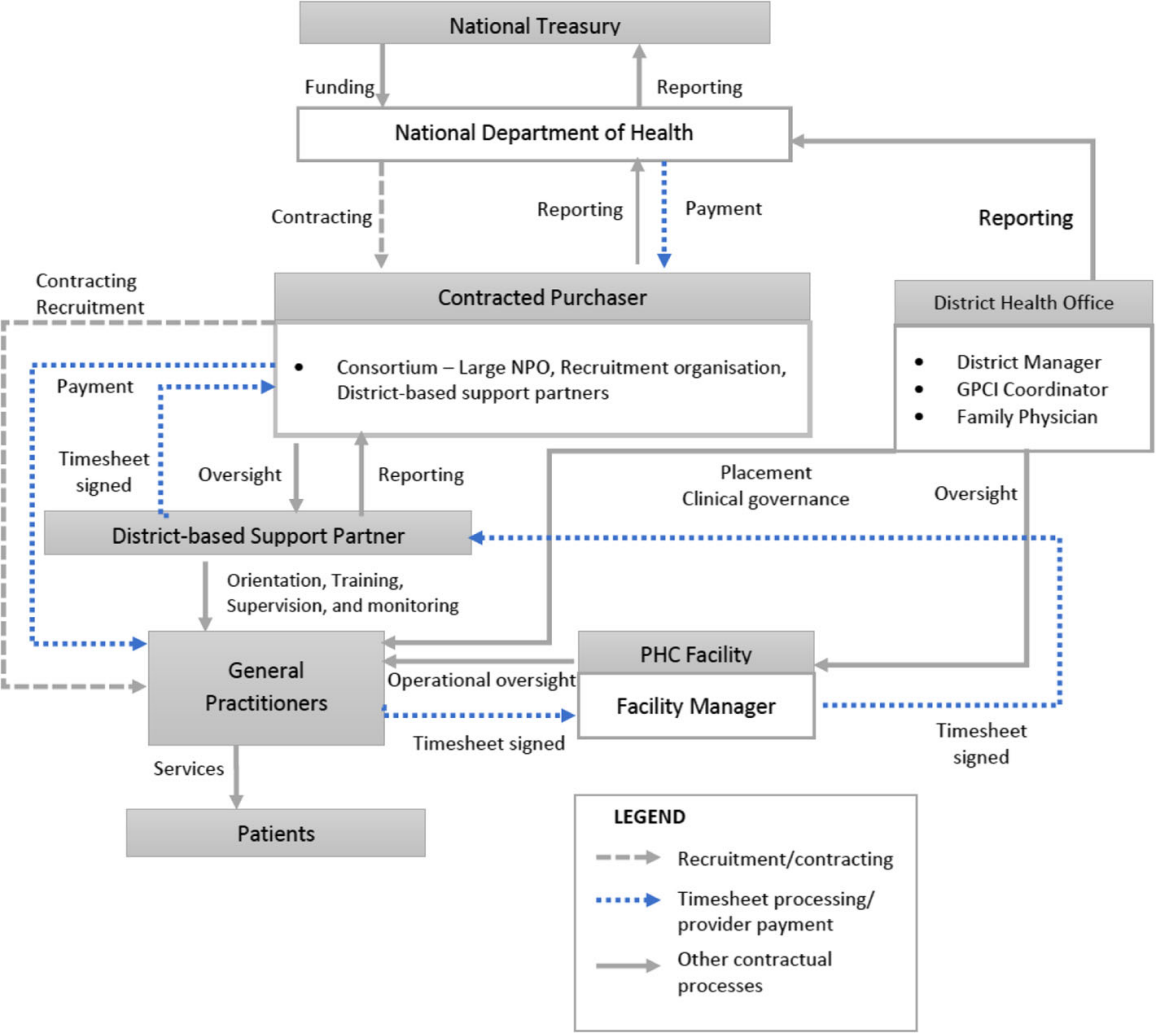
Contracted-purchaser model.The purchaser is an independent Service Provider (SP) contracted to manage implementation of the GPCI on behalf of the MOH. The SP - a large South African health not-for-profit organization with a national footprint – sub-contracts a variety of organizations which assume different roles in the contract management process. These organizations act as a Consortium, which is responsible for advertisement, recruitment, contracting, supervision, monitoring and payment. Recruited GPs are contracted directly by the SP, and their contracts are signed by the GP and an authorized signatory of the SP. Once a GP is appointed, the SP liaises with the DHO to determine a facility for placement. A district-based support partner (DSP) in each district – a sub-contracted district-based organization which is funded to support local health system strengthening – is then responsible for orientation, training, supervision, monitoring and performance management of the GPs. At a facility level, the FM is responsible for overseeing daily activities. GPs are paid monthly upon submission of a timesheet that is verified and co-signed by the FM, an authorized representative of the DSP and the SP’s project manager at the national office. The timesheets are then submitted to the SP’s finance department for verification and payment. The SP submits monthly and quarterly performance reports to the NDOH

**Fig. 3:**
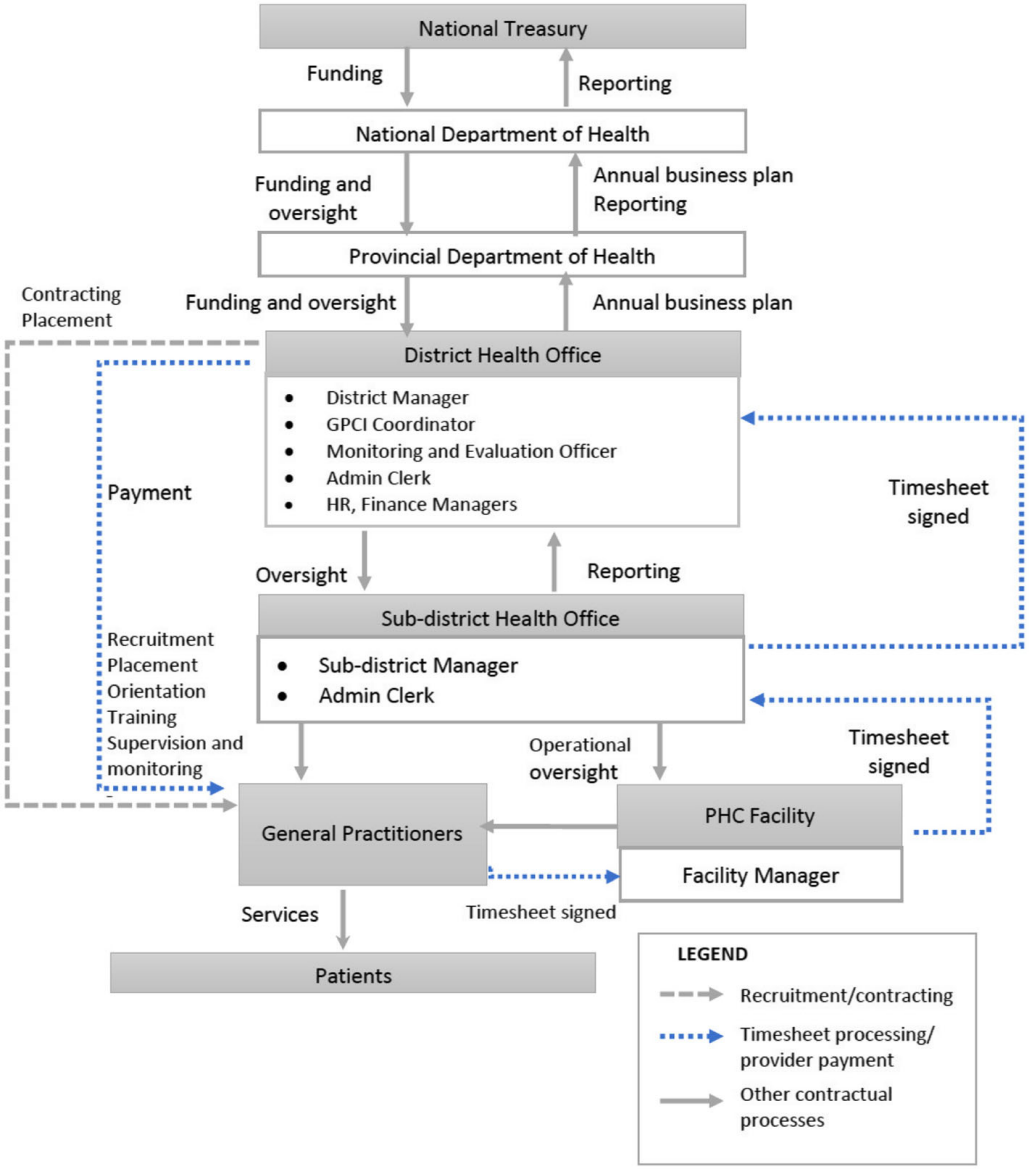
Decentralized-purchaser model.The provincial department of health (PDOH) is the purchaser. The GP enters a contract with the PDOH represented by the DHO, and the contract is signed by the GP and DM (as a representative of the PDOH). Recruitment and placement are done by the sub-district health office in conjunction with the DHO. The sub-district manager (SDM) is responsible for orientation, training, supervision and monitoring of GPs. GPs provide PHC services to patients attending PHC clinics with day-to-day oversight from a Facility Manager (FM). GPs are placed on the DHO payroll and paid at the end of the month based on the number of hours indicated in the contract. The GPs complete monthly timesheets that are in turn verified and signed by the FM, SDM, GPCI Coordinator and DM. These timesheets are not used to effect payment, but rather as an oversight mechanism to confirm the number of hours worked. The sub-district health office compiles and submits monthly and quarterly reports to the DHO and PDOH for review and submission to the NDOH

In this section, we begin by describing the three models of contracting-in (i.e. cases) in terms of how they function (Figs. 1, 2, and 3). Applying Liu’s conceptual framework on contracting-out, we compare and contrast characteristics of contractual arrangements in the three models. Table 4 presents a timeline of key events in the conceptualization and implementation of the GPCI including the release of key policy documents that underpinned the development of this initiative. We then describe the inception and early emergence of the GPCI in SA at a national level through the lens of the health policy analysis triangle. We conclude by exploring three key factors underpinning the characteristics and evolution of the three contracting models using the lens of the health policy analysis triangle and Liu’s conceptual framework.

**Table 4:** Timeline of key events in emergence of GPCI (2010–2017)

Year	Event	Key actors involved
1994	African National Congress Health Plan released	African National Congress (ruling party)
1997	White Paper for Transformation of the Health System released	Ministry of Health
2003	National Health Act (2003)	Ministry of Health
2010	PHC re-engineering discussion document released	Ministry of Health
August 2011	NHI Green Paper released	Ministry of Health
March 2012	10 NHI pilot districts announced	Ministry of Health
April 2012	NHI piloting in 10 selected districts commences	
2011–2012	National Technical Task Team (NTTT) constituted to drive GP contracting initiative	National policymakers, NTTT, provincial, district NHI coordinators, academics, representatives of professional associations
2012–2013	Policy intent and contracting model finalized (centralized-purchaser model)	National policymakers
	Ministerial roadshows held to engage with relevant stakeholders and promote buy-in for NHI and the GPCI	Minister of Health
February 2013	PDOH announces intention to pursue decentralized-purchaser model in the selected pilot district	PDOH, District level managers
2013	Centralized-purchaser model in implemented in selected districts Independent payroll company contracted to manage the payroll process at national level (centralized-purchaser model)	National policymakers, provincial, district level managers, facility managers GPs National policymakers
July 2013	Decentralized-purchaser model is implemented in one pilot district	Provincial, district and sub-district managers, facility managers, GPs
Early 2014	Decision taken by NDOH to pursue a contracted-purchaser model	National policymakers
November 2014	Service provider appointed and contracted to recruit, place and manage GPs (contracted-purchaser model) Contracted-purchaser model is implemented in selected districts	National policymakers
December 2016	Draft NHI White Paper is released for public consultation	Ministry of Health
June 2017	NHI White Paper is released	Ministry of Health

Source: Document review and interview data

### Three GPCI contracting-in models

This section describes each of the three contracting-in models, to which we have assigned the following nomenclature (1) a centralized-purchaser model, (2) a decentralized-purchaser model and (3) a contracted-purchaser model. For each model, we describe the purchaser, contractual processes and main actors involved. Key areas of variation in the models are: (1) The purchaser – the party with whom the GP has a contractual agreement with; (2) level of involvement of national, provincial and district actors in the contractual processes such as recruitment, training, supervision and monitoring of services (Table 5); and (3) the mechanism through which funds are channeled from the purchaser to the provider. All three models derive funding from the same source, namely the national government.

**Table 5:** Involvement of actors in contractual processes, by level of health system and case

Model	Centralized-purchaser model						Decentralized-purchaser model						Contracted-purchaser model					
Level of health system	N	P	D	SD	F	O	N	P	D	SD	F	O	N	P	D	SD	F	O
Aspect of contractual process																		
Recruitment	✓		✓						✓	✓			✓					✓
Contracting	✓								✓									✓
Placement			✓						✓	✓					✓			✓
Orientation and training			✓							✓								✓
Supervision and monitoring			✓		✓					✓								✓
Timesheets			✓		✓				✓	✓	✓						✓	✓
Payment	✓					✓			✓									✓
Reporting	✓		✓					✓	✓	✓			✓					✓

*N* National, *P* Province, *D* District, *SD* Sub-district, *F* Facility, *O* Other (External service provider)

### Centralized-purchaser model

Figure 1 depicts the centralized-purchaser model. The National Department of Health (NDOH) is the purchaser and directly recruits and contracts the GPs. Other than recruitment, contracting and high-level oversight, involvement of national actors is minimal. Provincial actors appear to be largely absent from the implementation process in this model. On-the-ground oversight and monitoring occurs primarily at the district level. The district health office (DHO) is responsible for GP placement, orientation, training, supervision and monitoring of service provision. Actors involved in activities at this level typically include the district manager (DM), a GPCI coordinator, a family physician and an administration clerk. The GPCI coordinator is usually a clinician manager who also has other responsibilities within the district (such as overseeing other doctors working within public sector health facilities). This is the primary individual responsible for overseeing all GPCI activities within the district. Other individuals within the DHO provide support as determined by the DHO management team. Payment to the GPs is effected monthly by an external payroll company upon submission of approved timesheets.

### Contracted-purchaser model

Figure 2 illustrates the contracted-purchaser model in which the purchaser is an independent Service Provider (SP) contracted to manage the implementation of the GPCI on behalf of the MOH. The SP is responsible for advertisement, recruitment, contracting, supervision, monitoring and payment of the GPs.

Unlike in the centralized-purchaser model, where the DHO is responsible for oversight at district level, in this model oversight is done primarily by a district-based support partner (DSP). The DSP is a sub-contracted district-based organization that is funded to support local health system strengthening. The DSP is responsible for orientation, training, supervision, monitoring and performance management of the GPs. In this model, the DHO’s involvement is limited to determining placement at a facility level. The DM, GPCI coordinator and family physician may liaise with the DSP on matters of clinical governance or as the need arises. As with the centralized-purchaser model, GPs are paid monthly on submission of a completed verified timesheet.

### Decentralized-purchaser model

Figure 3 illustrates the decentralized-purchaser model, in which the provincial department of health (PDOH) is the purchaser using funds disbursed by the National Treasury (NT). Provincial and district health departments develop annual business plans that outline the proposed total number of hours contracted GPs will work, including a budget and monitoring framework against which performance is measured. The business plan requires approval from the NDOH annually. GPs are contracted directly by the PDOH, represented by the DHO.

Actors involved in implementation within the DHO include the DM, GPCI coordinator, administration clerk, monitoring and evaluation officer, human resource and finance department staff members. At the inception of this model, the DHO led recruitment, placement, orientation and training of GPs with input from the sub-district manager (SDM). The DHO and the SDM were responsible for placing the doctor at an appropriate PHC facility. Supervision and monitoring were then conducted by the SDM. Over time, as the SDMs adapted to the process, most activities were transferred to the SDM with oversight from the DHO.

In contrast to the other two models, in the decentralized-purchaser model, GPs are paid in the same manner as public sector-employed doctors working in the district. The contracted GPs are placed on the DHO payroll and paid at the end of the month based on the number of hours indicated in the contracts. Timesheets are an oversight mechanism to confirm the number of hours worked but are not used to effect payment. The DM has the authority to stop monthly payments if a doctor does not work the number of hours required in the contract.

### Common features

In all three models, placements are determined by the DHO based on service needs and the FM is responsible for overseeing daily activities at the facility. Monthly and quarterly reports are submitted to the NDOH as part of the monitoring process. With the exception of the decentralized-purchaser model, provincial actors are minimally involved in contract management and oversight.

### Characteristics of contractual arrangements of the three models

Aspects of the contracts’ characteristics evolved as the GPCI models did. Thus despite the similarities among the models, distinct differences have emerged. Based on Liu’s framework we outlined the features of the purchaser and provider types, and each type’s financial and managerial capacities across the three models (Additional file 1). Table 6 outlines the characteristics of the contractual arrangements in each model, as per Liu’s framework. In all models, GPs are expected to deliver the full spectrum of PHC services and adhere to local public sector guidelines and practices. Their other functions include clinical mentoring, training and support of other health care workers within the facility, as well as clinical governance and quality assurance (QA). Under the contracted-purchaser model, the QA functions are better defined and the GPs are expected to participate in facility-based QA activities. All three contracting models are formal and legally-binding in terms of formality; the contracted-purchaser contract outlines performance requirements in the most detail and is therefore the most classical of the three contracts. Of note, however, the decentralized-purchaser model also has aspects that are more relational due to its decentralized management and because the local purchaser has built trust with the GPs over many years. Performance monitoring under the decentralized-purchaser model is also done at a sub-district level. Importantly, although the Ministry’s intention was to have a classical complete and legally-binding contract, this has proven challenging to enforce. At present, performance management of individual GPs is restricted to monitoring attendance as prescribed in the contract. Monitoring the quality of services has yet to be implemented except in the contracted-purchaser model.

**Table 6:** Characteristics of contracts by case

	Centralized-purchaser model	Decentralized-purchaser model	Contracted-purchaser model
Type of services	• Provide the full range of PHC services adhering to PHC guidelines and essential drugs list (EDL) at PHC facilities.		
	• Clinical mentoring, training, support and capacity building of other health care workers at the PHC facility.		
	• Provide oversight to PHC facility staff with regards to clinical governance and quality assurance.	• Provide oversight to PHC facility staff with regards to clinical governance.	• Quality assurance within PHC facilities through: performing clinical file audits, data reviews, monitoring supply chain management and equipment issues. • Participate in infection prevention and control activities in PHC facilities.
Contract formality	• Intent was to have a classical, complete and legally binding contract. • Monitoring performance and behaviours of GPs has been difficult and costly to enforce. • Centralised purchaser (NDOH) unable to monitor GPs directly. This monitoring function delegated to DHO. However, DHO feels unempowered to exercise this authority effectively due to nature of contractual relationship (DHO not direct purchaser).	• Contract is classical, complete and legally binding. • Some aspects lean towards relational type of contract (due to decision-maker preferences)	• Contract is the most classical, complete and legally binding of the three.
Contract duration	• Duration varies: 6 month, 1 and 2 year contracts; linked to funding availability which is received from NT on an annual basis. • Modified over time as the GPs were initially not willing to sign contracts of shorter duration.	• Annual. Aligned to an annual business plan, and is provided annually by the NDOH, which in turn is received from NT on an annual basis.	• Duration varies: one or two year contract, which is aligned to contracted purchaser’s contract with NDOH.
Provider selection	• Advertisements stipulating requirements placed.	• Advertisements stipulating requirements placed. • Intent was to have a competitive process.	• Advertisements stipulating requirements placed.
	• Not clear if all candidates appointed following interview process.	• Candidates appointed following interview process.	
	• Possibly influenced by contextual factors and supply of doctors.		
Specification of performance requirements and monitoring	• Performance requirements limited to delivery of services, timely submission of complete and quality timesheets and minimal or no incidents or default or breach of contract. • No specification of performance requirements relating to monitoring provision of clinical services, clinical governance or quality assurance.	• Performance requirements are specified in detail in the job description. • These cover four areas: (1) provision of clinical services, (2) clinical governance, (3) mentoring and support of other facility staff and (4) administrative tasks (completion of registers, timesheets and claims). • Specification of monitoring not clear. Performance monitoring done informally with exception of monitoring of administrative tasks.	• Performance requirements are specified in detail in the job description. • These cover five areas: (1) provision of clinical services, (2) staff and personal development, (3) facility quality improvement, (4) infection control and (5) finances (correct and timely timesheet and leave submissions). • Performance monitoring is done formally every six months. • GPs are required to maintain a detailed portfolio of evidence in support of their performance.
Provider payment mechanisms	• Completed monthly timesheets signed by GP, facility manager, GPCI district coordinator and district manager are required for payment to be effected monthly. • GPs are required to sign daily attendance registers at the facility, which is used to verify hours worked on the timesheet. • Payments were initially done through the NDOH finance department, and were later outsourced to an external Contracted Payroll Company. • Remuneration rate: R438 per hour (2016/17, exchange rate 1 USD = R14.7)	• GPs are placed on the district HR payroll and paid a fixed monthly amount. • Payments are executed by the district finance department. • The daily facility attendance register and completed monthly timesheets are signed by the GP, facility manager, sub-district manager, GPCI district coordinator and district manager used as verification mechanism. • If the GP does not work the required hours, the district manager has the authority to stop the monthly payments. • Remuneration rate based on years of experience. Between R180 and R312 per hour (2016/17).	• Completed monthly timesheets signed by GP, facility manager, district support partner and project manager required for payment to be effected monthly. • GPs are required to sign daily attendance registers at the facility, which is used to verify hours worked on the timesheet. • Payments are executed centrally by SP. • Remuneration rate: R500 per hour (2016/17).

### Inception and early emergence of the GP contracting-in initiative at a national level

This section describes the early emergence of the GPCI using the health policy analysis triangle as a lens. The inception of the GPCI can be traced over a seven-year period (highlighted in Table 4, which presents events in the introduction, emergence and early implementation of the initiative). The early emergence and establishment of the three models can be traced over a period of 4 years (2011 to 2014), between the release of the NHI Green Paper and initial implementation in selected NHI pilot districts.

#### Context

The first component of the health policy triangle model is the context in which it is exists. Beginning in 1994, the newly elected political establishment set out to address the legacy of the inequities of apartheid. A series of high-level policies were outlined in key policy documents and legislation, including the White Paper for Transformation of the Health System (1997) [35], PHC re-engineering document (2010) [36], Negotiated Service Delivery Agreement (2009) [23] and National Development Plan (2011) [37]. These efforts sought to restructure and unify a previously fragmented public health sector, expand access to health care, and improve health system management. Implementing NHI as a financing mechanism to enable achievement of UHC has been a cornerstone of the ruling party’s political manifesto since it came into power in 1994 [38]. Between 1994 and 2009, a series of high-level committees were set up to investigate the feasibility of introducing a NHI programme. Their findings paved the way for the development of the NHI policy documents (NHI Green and White Papers) [4, 5, 22]. High-level political will to address past inequities helped propel NHI onto the policy agenda, as it represented one of the mechanisms to redress structural imbalances. Reforms were centered around a renewed commitment to PHC and a shift in health service provision focus from a hospital-centered model to a more preventive and PHC-oriented approach.

GP contracting-in is considered the fourth arm of the PHC re-engineering strategy, which aimed at strengthening the PHC platform. This included focusing on increasing access to and coverage of PHC through drawing on the pool of private GPs who typically serve the smaller insured population and are primarily located in urban areas. The intended recipients of health services provided by the contracted GPs were patients accessing public sector PHC clinics in rural or semi-rural districts.

#### Actors

The second component of the health policy triangle is the actors involved. Conceptualization of the GPCI was primarily a nationally-driven process. The development of the NHI policy and the GPCI was largely driven by high-level policy elites, including the Minister of Health. A National Technical Task Team (NTTT) for GP contracting was set up in 2011 following the initial NHI Green Paper. Chaired by a high-level national policymaker, the NTTT included other high-level national policymakers, provincial and district level managers and NHI coordinators. NHI coordinators were appointed by the MOH to provide oversight for all NHI implementation initiatives, including the GPCI. Other NTTT participants included academics and representatives of professional associations. Some interview respondents suggested that the representatives of professional associations may not have been representative of all GPs or that information from the negotiations did not cascade down to all GPs; these gaps are evident in subsequent dissatisfaction with the remuneration rates. Given the intention to address inequities, policymakers also included representatives from a rural health advocacy group to ensure consideration of rural contexts.

*“We also have a task team that is set for the GPs, because remember when we implemented this [GPCI]..…no structures existed.…In terms of the [National Treasury] grant framework, we had to ensure that we had a technical task team that would look at the GP contracting. We would look at implementation, M&E, any issues, anything regarding the GPs, we would then handle it in that sort of task team.”* (National level manager 2).

Despite its attempts to be inclusive, facility managers and GPs appear to have been largely absent from this initial process. Neither the criteria for inclusion in this NTTT nor the process of its constitution were made clear in the interviews. The high-level policymakers were able to exercise their power in the process through decision-making at various points in the policy development process. These included among others setting the policy agenda, constituting the NTTT and overseeing funding disbursements. This largely top-down policy process may have resulted in the lack of a sense of ownership by the GPCI implementers and providers, eventually leading to implementation gaps.

#### Policy content

The policy’s content forms the third component of the triangle. The NHI Green Paper provided early guidance in terms of preparation of the health system for eventual introduction of NHI. It proposed that contracted private practitioners deliver PHC services within a specific district and suggested establishing a District Health Authority to be responsible for contracting with the NHI to purchase the services of private providers [4]. Importantly, the decision to choose a contracting-in as opposed to contracting-out model was driven by various factors. As noted, a key intention underpinning the initiative was to improve access to PHC services particularly in rural and geographically remote areas. Our interviews confirmed that the lack of human resources, specifically doctors in rural clinic settings, and overburdening of hospitals contributed to the conceptualization of the GPCI in its current form. Another intention of the GPCI was to rouse confidence in the public sector by ensuring availability of doctors at health facilities. One interviewee described the intention thus:

“*So the purpose of this [GPCI] was to test modalities for gaining better access to doctors at PHC level, thus improving the quality of clinical care at PHC level and increasing patient confidence in PHC services. So, to stop the bypassing of PHC facilities to hospitals because hospital services are more expensive services, so you go towards implementing the NHI.*” (National level manager 5).

Further, the public sector is primarily a nurse-led system. Contracting-in doctors was also considered to be a way that would not only provide much-needed services, but would also provide support to overwhelmed PHC nurses through mentorship and access to immediate referral pathways. One respondent commented:

“*So the one [benefit] would be quality assurance, teaching and training, referral for those patients who need to be referred and be appropriately seen by a doctor, and injecting confidence into a nurse-based primary health care system.*” (National level manager 4).

Interviewees reported that during initial discussions both contracting-in and contracting-out options were considered. The potential challenges with monitoring outputs and quality of services provided by doctors in their own practices led to the choice of contracting-in. The contracting-in option thus also sought to ensure that GPs would follow national treatment guidelines and policies, rather than subsidizing non-standardized care in their own private practices, as two respondents highlighted.

*“We had a very intense debate at the early days of whether it would be contracting-in or contracting-out. I recall meeting with [high-level policy officials], and in the first meeting of the GP contracting task team, the very first was…speaking about both models [contracting-in and contracting-out].”* (National level manager 9)

“*Quality [of services] in GP practices pervasively is not what we expected to be either, and many of the GPs in SA are out of touch with clinical guidelines as developed….If we had contracted out, you simply send your patients to the doctors to do what they always do. But bringing the doctors into the public sector facilities was also a way of getting them to conform to public sector guidelines.”* (National level manager 5).

Although the intention to pilot contracting of GPs was outlined in the NHI Green Paper, at the time the study was conducted there was no policy document available specific to the GPCI that outlined the content, processes and actors to be involved in implementation, as one respondent mentioned.

*“I can’t remember if I saw a document specifically outlining [GPCI]. I think it was more in discussion.”* (District A manager 6).

Documents such as contract templates, timesheets and monitoring reports were nonetheless available.

As the GPCI is an NHI pilot initiative, it receives funding from a single source: an NHI conditional grant from the National Treasury (NT) that was created to support the first phase of the NHI roll-out in 2012/13. Conditional grants are financial allocations from the NT to either national or provincial government departments that may only be used for a specific designated purpose. Funds are therefore administered by the NDOH and need to comply with stipulated reporting requirements. With the exception of the decentralized-purchaser model, provincial health departments have little to no autonomy in managing funds disbursed for the GPCI. GPs contracted into the services were to be paid a specified fee-per-hour (session) worked in a facility. The number of hours a GP works at a facility can differ, determined by the need in the facility as well as the time the doctor has available. The number of hours the GP worked was to be negotiated between each GP and the purchaser.

#### Policy process

The final component of the health policy triangle is the process by which policies are elaborated. Following the release of the NHI Green Paper, eleven pilot districts were selected where innovations for health system reform, such as GPCI, would be tested and evaluated. The NTTT’s initial purpose was to deliberate the pros and cons of various contracting options, and to conceptualize a contracting model to pilot. Once the model of contracting-in was decided on, the NTTT became responsible for monitoring implementation against set targets and providing timely feedback on the process as it occurred.

*“When it [NTTT] started, it was a monthly meeting. So they [NTTT members] were coming to report and then we’ll agree on things that are not going well and trying to make sure things are working. So that body (or that task team) was making sure things are being implemented the way they are supposed to be implemented.”* (National level manager 1).

Concurrently, the Minister of Health embarked on a series of “national roadshows” (or public campaigns) in the selected pilot districts aimed at raising awareness around NHI and garnering interest in the GPCI among local GPs. Targeted participants in these activities included district and provincial managers, GPs and other health practitioners, local councillors, NGOs, academics, and representatives of professional associations.

### Factors influencing emergence of the three contracting models

In this section we reflect on the factors that influenced the emergence of the three GPCI models during the early implementation phase. The three main factors we identified were: (1) the decision to pilot the implementation of the GPCI; (2) the financial management capacity; and (3) the managerial capacity of the national, provincial and district actors involved in implementation.

#### Piloting as the model for early implementation

The decision to pilot implementation of the initiative was a key factor facilitating the emergence of the three GPCI models. Piloting allowed for flexibility in the implementation process, enabling course corrections in response to challenges that emerged during the early implementation phase. One respondent described piloting as a fluid process, with changes made depending on what worked and what did not.

*“As a pilot you want to know what will work what will not work. We needed to start somewhere. Then after starting at that point, there will be some suggestions coming in and then we will change as it comes. Because if people are coming with suggestions, why not change? If there is something that is not working, why not drop it? So that’s how it worked, and even that’s how it is still working. So if you see that something was not part of the plan and we think it will work, why not include it?”* (National level manager 1)

The flexibility enabled through piloting also created greater scope for provincial autonomy, thereby facilitating the emergence of the decentralized-purchaser model. This was further legitimized by existing legislation that enabled a level of provincial-led decision-making. High-level provincial and district actors in the decentralized model decided to build on their existing local model of contracting non-state providers rather than opting for the nationally-driven contracting model. Thus following the initial ministerial roadshows, the proposed contracting-in model was adapted to suit the local context and experience, while complying with the reporting requirements stipulated by the funding source. Building on existing local models and approaches also facilitated the emergence of a more relational contract in the decentralized model, in which mutual trust is a central principle. Provincial leaders also saw this as an extension of their existing engagement with private sector providers. The decision to adopt decentralized models was thus made at the outset of the GPCI and the province never adopted a centralized version of the GPCI.

Flexibility during piloting also resulted in changes to the contractual characteristics. For example, changes to the provider payment mechanisms in the centralized and decentralized purchaser models were made to ensure timely payments to providers. Although driven by other factors related to financial and managerial competencies (discussed below), the decision to pilot enabled easier implementation adaptations.

However, despite the flexibility created by piloting, a few respondents felt piloting might have been inadequate and that more emphasis could have been placed on testing alternative contracting models during this early implementation phase. In particular, different contractual characteristics could have been tried.

*“Piloting has a particular meaning and we haven’t actually piloted anything yet, in the real sense of piloting. Piloting means testing new concepts and ideas and evaluating them on small scale for potential scaling up….It’s testing these approaches – of capitation, of pay-for-performance and the like.”* (National level manager 3)

*“It [GPCI] was introduced as a concept to be piloted and I think it did serve its purpose as a pilot to demonstrate what the problems were and where the problems were and what are the things we need to do to change.… At this stage, I would say it can no longer be considered a pilot. It should be considered as a policy direction, where we identified where the challenges are. And we are now implementing it with lessons learnt from the pilot.”* (National level manager 4)

#### Financial management capacity

GPCI’s reliance on a centralized funding source – the NHI conditional grant administered through the national MoH – resulted in the development of stringent mechanisms for monitoring GPs’ attendance at clinics, including registers and timesheets, to ensure compliance with NT reporting and payment mechanisms.

At the inception of the centralized-purchaser model, all payments were processed by the finance unit within the national MoH. However, lack of financial management capacity and bureaucratic inefficiencies led to delays in payments, and this function was eventually outsourced to an independent payroll company.

*“It was initially done by NDoH and that’s when there was a problem and then that’s when we contracted [payroll company]….But they [payroll company] didn’t start with it [GPCI]. They came later due to challenges that we were having at the [national] department.”* (National level manager 1)

Within the decentralized-purchaser model, the provider payment mechanism was adapted to match local financial practices and past institutional (provincial and district) experience with paying contracted providers. GPs were placed onto the district payroll and paid a specified monthly amount based on the number of hours stipulated in their contracts. Monthly timesheets were used to verify hours worked. The decision to place the GPs on the payroll and to pay them monthly was aimed at improving the payment process and easing the financial management burden on the district staff. More specifically, district management sought to eliminate the risk of late payments which could occur as a result of the time it took to sign the timesheets in order to effect payments, and the possible negative effect this could have on their relationship with local GPs.

#### Managerial capacity

At GPCI’s inception, the choice of contracting-in (versus out) of GPs was linked to the need to closely monitor the quality of services provided to patients accessing public sector PHC facilities. The contract design, and the choice of monitoring through monthly timesheets were intended to ensure that the state could monitor outputs and control provider behavior by only paying GPs for hours worked.

“*No it wasn’t just logistical, it was a fear that the department will not be able to manage the risk of any fraudulent activity.” (National level manager 3)*

Requiring contracted GPs to follow the public sector’s Standard Treatment Guidelines was also intended to ensure their integration into existing service delivery platforms.

Another key factor that drove the emergence of the contracted-purchaser model was the managerial challenge faced by the Ministry in all areas of the GPCI: recruitment, contracting, training, monitoring and supervision of the GPs. As the MoH was unable to manage these processes and meet recruitment targets, outsourcing these functions to an external SP represented a good alternative.

“Clearly I think there were insufficient people here [at national level] to deal with it [GPCI], in terms of administration, for contracting and all of that.” (National level manager 3)

Both province and district systems endeavored to integrate the GPs into the existing service delivery platform. The decentralized-purchaser model emerged in response to the provincial authorities’ need to more tightly control the GPCI. The provinces’ prior experience in managing external providers was also a motivating factor, and systems were already in place to effectively manage these GPs.

*“We just thought that it was better to have control, who works where, and what they should do, rather than having an external driver doing the contracting. And as they explained the previous experience…we just wanted the full control over the process.”* (District A manager 5)

## Discussion

The three GPCI models described emerged from an initiative that piloted contracting GPs into the public health service in SA. The models represent three different types of purchasers: (1) the central Ministry of Health, which directly contracts GPs and manages the contract; (2) a contracted SP that reports to the Ministry while directly contracting GPs and sub-contracting with a variety of organizations that assume various roles (e.g. recruitment); and (3) a province that has decentralized the contracting process to the district and the sub-district levels, while providing high-level oversight.

Our study aim was to draw lessons for future policymaking and health system strengthening to attain UHC by using NHI as the financing mechanism. This paper’s exploration of the three GPCI pilot models reveals important lessons. First, piloting promoted flexibility in implementation and allowed the GPCI to be shaped by the various contexts and actors to meet local needs. While contract characteristics should be well-defined, implementation should be flexible and tailored to the local setting. Among the study respondents, however, discordant views were expressed about the adequacy of piloting of the initiative, with some describing piloting as ongoing and others voicing the need to further test out alternative models. This may point to a lack of consensus among the actors on the nature and degree of piloting. It also resonates with the recommendation by Heard et al. to pilot contracting on a “meaningful scale” to build experience and capacity within the government with the ultimate aim of improving engagement with non-state providers [39].

Second, flexibility creates opportunities to recognize and enable local capacity to effect policy implementation. The evolution from the initial centralized-purchaser model to a contracted-purchaser model, due to human resource limitations and financial management challenges, is one example. This created opportunities for the SP and its consortium of organizations to assume a primary purchasing role. Third, management capacity was a key factor influencing the emergence of the GPCI models and subsequent implementation. The importance of management capacity in contracting with non-state providers in LMICs has likewise been highlighted in previous studies [31, 40–42]. Where capacity existed and policy actors leveraged the province’s autonomy to implement the decentralized-purchaser model, decentralized GPCI management was selected as the approach. Importantly, this decision appeared to be largely driven by existing institutional experience and systems for non-state provider contracting, and thus the province’s confidence that this could be best managed locally. This points to the importance of delegating decision-making and empowering actors at a local level and emphasizes the interaction between institutional capacities, decision space and accountability, as suggested by Bossert and Mitchell [43].

A notable area of uncertainty is the role played by provincial health departments in the emergence of the GPCI. With the exception of the decentralized-purchaser model (where provincial-level actors and processes played a critical role in enabling and facilitating a decentralized model), provincial health departments were minimally involved in the evolutionary process. Given the structure of the South African health system, as well as the legislated autonomy of the provinces, their absence from the GPCI’s development has implications for future buy-in and sustainability of the GPCI, and other efforts to contract private providers into the public sector. Provincial roles are however not clearly articulated in the White Paper [22], with most responsibilities and activities proposed to be located at the district level.

In this paper we did not set out to make judgements on the success or failures of the various models or their evolutionary processes– an assessment of the implementation of the GPCI pilot will be presented elsewhere. What we sought to illustrate was that establishing this form of engagement with non-state providers: (1) is a large and resource-intensive undertaking; (2) must be determined by local context; (3) needs to account for a people-centred approach to health care; and (4) requires significant financial and general management capacity, resources and experience.

Lessons gleaned from the GPCI’s evolution will be useful as SA includes non-state PHC providers into the public sector realm in the ongoing drive to implement NHI. The recently-released White Paper for NHI in SA articulates a vision of a publicly-administered National Health Insurance Fund (NHIF) that is a strategic single-purchaser, a single-payer and reports to the Minister of Health. This includes a specific contracting unit to be located within the NHIF. Both contracting-in and out will be options for engaging private practitioners to work in PHC settings to provide services based on need. These providers will be remunerated on a risk-adjusted capitation basis, frequently evaluated and monitored and receive additional remuneration based on performance. Practitioners will be expected to meet appropriate professional requirements as a prerequisite to being contracted. The White Paper further envisages that a Contracting Unit for Primary Health Care (CUP) will be located at district level to contract and manage GPs. Importantly, the White Paper expresses the government’s commitment to test out various implementation approaches and learn from these activities [22].

Lessons learned from this study suggest that as NHI is rolled out, the SA National government should implement and test contracting approaches. The evidence presented here suggests that flexibility and tailoring to local contexts and capacities is beneficial, and that a one-size-fits-all approach should not be considered. This study also supports a more decentralized, as opposed to centralized, approaches.

Further, to implement closely-monitored GP contracts using decentralized CUPs will undoubtedly require well-resourced services, well-functioning systems, and capacitated staff. Given the importance of management capacity on the emergence of GPCI, an a priori assessment of the state’s management capacity should be an integral part of any future contracting initiatives with non-state providers (NSPs) [42]. Successful contract management has been linked to external management support [44], suggesting that provision of additional management support should be a consideration for future contracting initiatives. This study supports this, highlighting the importance of strong oversight such as a strong provincial team that supports lower-level implementation and facilitates decision-making as required. The national Ministry of Health should therefore ensure that local-level administrative structures are ready to implement and relevant staff are appropriately capacitated. Thus flexibility and willingness to implement a contracting model when districts are fully capacitated are paramount.

### Strengths and limitations

This is one of the first studies to describe the GPCI models in detail and chart their emergence at a national level. Its strength lies in the inclusion of multiple perspectives, including national, provincial and district managers, independent service providers and GPs. This enabled triangulation of the data from document reviews with interviews from actors involved in the initiative at various levels of the health system.

Nevertheless, we acknowledge several limitations of the study. First, the study only included three districts. Thus it may not be appropriate to generalize our findings to other districts in SA, which has diverse regional contexts. Nonetheless, we found valuable insights on the GPCI emergence and implementation and believe it would be useful to utilize these when examining the initiative in other settings. Second, the inception of the GPCI occurred over a seven-year period (between 2010 and 2017) with the three models emerging between 2011 and 2014. During and since that time, the initiative has undergone changes in leadership at the national level and a lack of involvement of provincial managers in high-level policy decisions on the nature of contracting. Furthermore, few documents exist that articulate the policies. We were therefore unable to elucidate or verify some of the early events in the inception of the GPCI following the release of the NHI Green Paper and the decision to choose a contracting-in model.

Lastly, our study did not specifically aim to include facility managers as respondents. Future studies on subsequent implementation of the GPCI including the perspectives of facility managers could provide additional insights into on-the-ground implementation and interactions between GPs and other cadres of health care workers in SA’s traditionally nurse-led PHC facilities. Future publications emanating from the broader study of which this paper is a component will present factors that influenced implementation of the GPCI pilot to date.

## Conclusions

In summary, the three GPCI models that emerged essentially represented iterations of the centralized-purchaser model. Emergence of other two models were strongly influenced by purchasers’ capacity to manage contracts, payments and recruitment processes. Findings from the decentralized-purchaser model show the importance of local contexts, provincial capacity and experience in influencing the evolution of the models. Contract formality differed slightly by model, influenced by context and type of purchaser. Our key lesson is that even as contract characteristics need to be well-defined, adaptability to the local context and capacity is critical. Purchaser capacity, existing systems, institutional knowledge and experience in the area of contracting and financial management should all be considered before adopting a decentralized implementation approach to contracting with NSPs. These findings present important considerations for the future roll out and success of NHI in SA.

## Additional file

Additional file 1: Summary of the features of the purchaser and provider types, and each type’s financial and managerial capacities across the three contracting-in models. (DOCX 44 kb)

## Abbreviations

CUP: Contracting Unit for Primary Health care
DHA: District Health Authority
DHMT: District Health Management Team
DHO: District health office
DM: District manager
DSP: District-based support partner
EDL: Essential drugs list
ERC: Ethics Review Committee
FFS: Fee-for-service
FGD: Focus group discussion
FM: Facility manager
GDP: Gross Domestic Product
GP: General practitioner
GPCI: General practitioner contracting initiative
HPSR: Health policy and systems research
HREC: Human research ethics committee
KII: Key informant interview
LMICs: Low and Middle Income Countries
MOH: Ministry of Health
NDoH: National Department of Health
NHI: National Health Insurance
NHIF: National Health Insurance Fund
NPO: Not-for-profit organization
NSP: Non-state provider
NT: National Treasury
NTTT: National Technical Task Team
OOP: Out-of-pocket payment
PDOH: Provincial Department of Health
PHC: Primary Health Care
PMB: Prescribed minimum benefits
PTDS: Part Time District Surgeons
QA: Quality assurance
SA: South Africa
SAIMD: South African Index of Multiple Deprivation
SDG: Sustainable development goal
SDM: Sub-district manager
SEQ: Socio-economic quintile
SP: Service provider
UHC: Universal health coverage
