# Supplementary material for: Emergence of three general practitioner contracting-in models in South Africa: a qualitative multi-case study
Source: Int J Equity Health. 2018 Oct 5;17:107. doi: 10.1186/s12939-018-0830-0 (PMC6172712; doi:10.1186/s12939-018-0830-0)
Supplement: Supplementary file 1 — Summary of the features of the purchaser and provider types, and each type’s financial and managerial capacities across the three contracting-in models. (DOCX 44 kb) [file 12939_2018_830_MOESM1_ESM.docx]

**Additional file 1**

**Summary of purchaser/provider type and capacity per model**

|  | ***Centralized-purchaser model*** | ***Decentralized-purchaser model*** | ***Contracted-purchaser model*** |
| --- | --- | --- | --- |
| **Type of purchaser** | - NDOH is the direct, single purchaser (central). - NDOH contracts GPs directly. - GPs managed at district and facility level. | - District is the direct, single purchaser (local, decentralized) with provincial oversight. - District contracts GPs directly; - GPs managed at sub-district level with district oversight. Initially the district was more involved, but with time more oversight responsibilities were transferred to sub-district. | - NDOH contracts an external SP, who takes on purchasing functions. NDOH is therefore not the direct purchaser and plays a nominal role. - The SP (a centrally-based contracted purchaser) directly contracts the GPs on behalf of NDOH. - At district level GPs managed by a district support partner (sub-contracted by the SP). The latter form part of a broader Consortium, led by the SP. |
| **Purchaser financial capacity** | Partial   - Funding source for all models is a central NHI conditional grant from National Treasury (NT). - **Initially** the centralized purchaser (NDOH) had complete financial management responsibilities including timely payment of GPs. - **Later** (2014) contracted external SP to manage payroll due to inability to ensure timely payments to GPs. - There have been fluctuations in funding for GPCI through the grant. Stability of future funding not guaranteed. | Full   - Funds disbursed from NDOH as part of the conditional NHI grant. - Decentralized purchaser has complete financial management responsibilities including timely payment of GPs. - Purchaser adapted payment mechanism to decrease risk of untimely payments to GPs - Stability of future funding not guaranteed. Linked to reliance on central funding source that requires annual approval from NDOH. | Full   - SP receives funds from NDOH on submission of monthly invoice to NDOH. Funds paid to the SP are part of the conditional NHI grant. - SP pays GPs monthly on receipt of signed timesheets. - As purchaser SP has complete financial management responsibilities. Includes timely payment of GPs and Consortium partners. - As a SP to NDOH experiences challenge receiving timely payments from NDOH. Poses a financial risk to the contracted purchaser. - Stability of future funding not guaranteed. Linked to reliance on central funding, and contractual timeline with NDOH. |
| **Purchaser managerial capacity** | Partial:   - **Initially** centralized purchaser (NDOH) had complete managerial capacity over procurement (of services from GPs), oversight and implementation of payment systems. - The DHO has been responsible for day-to-day oversight and performance monitoring since inception. - Insufficient staff numbers (at national and district levels) and technical limitations have limited the centralized purchaser’s ability to procure services (contract GPs) and implement provider payment systems efficiently. - **Subsequently** the purchaser outsourced functions such as procurement, oversight, performance assessment and implementation of payment systems to an external SP through the development of the contracted-purchaser model. - Recruitment of GPs into the centralized-purchaser model was terminated in November 2014. - 62.3% of GPs in two of the three districts have remained on this model. - Centralized purchaser continues to be responsible for oversight, performance assessment and implementation of certain aspects of payment (timesheet review) for GPs who were initially contracted on this model. However, implementation of payment systems has been outsourced. | Full   - Working closely with the PDOH, the decentralized purchaser (district, including the sub-district for certain functions) has complete managerial capacity. This includes being responsible for procurement of services and implementation of payment systems. - Some aspects of recruitment, orientation, day-to-day oversight and performance assessment decentralized to the sub-district level. | Full   - Have complete managerial capacity including procurement (of services), oversight, performance assessment and implementation of payment systems. - Managerial responsibilities within the Consortium are cascaded. With the lead SP being responsible for managing its sub-contractees to assist with these management functions. - Sub-contractees (district support partners) in turn are responsible for day-to-day oversight of GPs, performance assessment, and implementation of payment mechanisms instituted by SP (reviewing and signing timesheets). |
| **Provider type** | Minimum requirements for participation are that GP be qualified as independent general practitioner and be registered with the Health Professions Council of South Africa [which requires that the doctor have completed a compulsory two-year internship and one-year community service in a rural hospital].  Policy intent was to contract private GPs (for profit), namely those who have their own practices. In reality, any medical practitioner who met the above requirements (except those full-time employed by the State) were contracted into the public sector. | | |
| **Provider capacity** | - All contracted GPs required to undergo standardized training to ensure they are up-to-date with public sector treatment guidelines. - GPs with prior public sector experience and younger GPs adapted well to public sector treatment guidelines. - Some GPs lacked administrative skills to fulfil administrative requirements of contract (e.g. completion of timesheets) timeously. | - All contracted GPs required to undergo standardized training to ensure they are up-to-date with public sector treatment guidelines. - GPs with prior public sector experience and younger GPs adapted well to public sector treatment guidelines. - Some GPs lacked administrative skills to fulfil administrative requirements of contract (e.g. completion of timesheets) timeously. | - All contracted GPs required to undergo standardized training to ensure they are up-to-date with public sector treatment guidelines. - GPs with prior public sector experience and younger GPs adapted well to public sector treatment guidelines. - Provision of quality assurance services challenging due to lack of time – GP felt outside scope of practice and time better spent attending to waiting patients. - Some GPs lacked administrative skills to fulfil administrative requirements of contract (e.g. completion of timesheets) timeously. |
